# Detection of Aβ plaque deposition in MR images based on pixel feature selection and class information in image level

**DOI:** 10.1186/s12938-016-0222-x

**Published:** 2016-09-15

**Authors:** Yongming Li, Xueru Zhu, Pin Wang, Jie Wang, Shujun Liu, Fan Li, Mingguo Qiu

**Affiliations:** 1 Department of Medical Image, College of Biomedical Engineering, Third Military Medical University, Chongqing, 400038 China; 2 College of Communication Engineering, Chongqing University, Shapingba District, Chongqing, 400044 China

**Keywords:** Alzheimer’s disease, Amyloid β plaque deposition, MRI, Detection, Pixel feature selection, Classification, Image level

## Abstract

**Background:**

Amyloid β-protein (Aβ) plaque deposition is an important prevention and treatment target for Alzheimer’s disease (AD). As a noninvasive, nonradioactive and highly cost-effective clinical imaging method, magnetic resonance imaging (MRI) is the perfect imaging technology for the clinical diagnosis of AD, but it cannot display the plaque deposition directly. This paper resolves this problem based on pixel feature selection algorithms at the image level.

**Methods and results:**

Firstly, the brain region was segmented from mouse model brain MR images. Secondly, the pixels in the segmented brain region were extracted as a feature vector (features). Thirdly, feature selection was conducted on the extracted features, and the optimal feature subset was obtained. Fourthly, the various optimal feature subsets were obtained by repeating the same processing above. Fifthly, based on the optimal feature subsets, the final optimal feature subset was obtained by voting mechanism. Finally, using the final optimal selected features, the corresponding pixels on the MR images could be found and marked to show the information about Aβ plaque deposition. The MR images and brain histological image slices of twenty-two model mice were used in the experiments. Four feature selection algorithms were used on the MR images and six kinds of classification experiments are conducted, thereby choosing a pixel feature selection algorithm for further study. The experimental results showed that by using the pixel features selected by the algorithms in this paper, the best classification accuracy between early AD and control slides could be as high as 80 %. The selected and marked MR pixels could show information of Aβ plaque deposition without missing most of the Aβ plaque deposition compared with brain histological slice images. The hit rate is over than 90 %.

**Conclusions:**

According to the experimental results, the proposed detection algorithm of the Aβ plaque deposition based on MR pixel feature selection algorithm is effective. The proposed algorithm can detect the information of the Aβ plaque deposition on MR images and the information can be useful for improving the classification accuracy as assistant MR biomarker. Besides, these findings firstly show the feasibility of detection of the Aβ plaque deposition on MR images and provide reference method for interested relevant researchers in public.

## Background

Alzheimer’s disease (AD) is a progressive, neurodegenerative disease that is characterized by severe deterioration in cognitive function, especially memory loss, and it is the most common type of dementia. Today, it represents a major public health problem and accounts for the majority of the whole population with dementia. An early diagnosis of AD will allow patients to benefit from effective treatments that can slow the neurodegeneration process [1]. Therefore, early diagnosis of AD is very necessary.

With the emergence of symptomatic treatment and the promise of drugs that can delay disease progression, the development of diagnostic biomarkers for AD has become very important. Relevant studies have shown that the β-amyloid (Aβ) protein is the main component of senile plaques. A marked increase in Aβ in anatomical structures (e.g., cerebrospinal fluid, CSF) in AD has been found in numerous studies. Importantly, increased Aβ has also been found very early in the disease process, before the onset of clinical symptoms [2]. Recent studies have suggested that Aβ has satisfactory performance when used as a diagnostic marker for AD in routine clinical practice [3]. Research has also shown that the main pathologic characteristic of AD is that Aβ deposition appears in the cerebral cortex and hippocampus, which gradually accumulate senile plaques (SPs). Previous studies have indicated that Aβ, which has strong neurotoxicity, is the core pathogenic substance of AD and is the most important prevention and treatment target for AD. Research has shown that Aβ begins to deposit 15–20 years before AD symptoms occur. Therefore, the noninvasive detection of the Aβ would be very helpful to the early diagnosis of AD. In addition to detecting Aβ plaque deposition, it is possible to increase the noninvasive early diagnostic accuracy in AD.

Currently, early diagnosis and treatment of AD based on Aβ have achieved encouraging results, including Aβ immune histochemical diagnosis, immune therapy, nerve factor therapy and so on [4–7]. However, due to technological deficiencies in the noninvasive detection of Aβ, it is difficult to attain the goal of clinical application of research for early diagnosis and timely intervention therapy. Therefore, it is very necessary and urgent to establish early, noninvasive detection technologies to detect Aβ in vivo [8].

Research studies have shown that positron emission computed tomography (PET) combined with a tracer, such as 11C-PIB, could display information about Aβ plaque deposition distributed in several anatomical structures, such as the cerebral cortex, white matter and so on [8–11]. However, the PET imaging method has the following disadvantages that prevent it from clinical application: (1) the tracer emits radiation, so patients are likely to oppose to this type of detection method; (2) PET essentially has low resolution and data volume, and it does not detail human physiological metabolism, so it cannot provide information about anatomical structures and small lesions, and it is unacceptable to clinicians; and (3) the price of PET is much higher than that of MRI, so patients tend to prefer latter to the former because dementias are chronic, non-lethal diseases [12–15].

Compared with the disadvantages of PET, MRI is inexpensive and noninvasive, it involves no radiation and no tracer, it has high resolution, and it has been widely applied in clinical applications. It can precisely and quantitatively reflect the changes in structure and function occurring in different brain tissues, and it has been widely applied for the early diagnosis of AD [16]. In recent years, studies have shown that β-amyloid plaque deposition could be reflected by MR without tracer under the high field intensity of MR because Aβ can absorb iron and calcium deposits [17]. MRI could reflect information about Aβ plaque deposition in the hypothalamus, hippocampus and cerebral cortex [17–19]. Insoluble cellulose caused by Aβ leads to the rapid attenuation of proton magnetization. Thus, on MRI, the brightness of relevant regions will decrease apparently, and the contrast will also change [20].

Based on the analysis above, although MR cannot display information about Aβ plaque deposition information directly, it can reflect information. Therefore, it is necessary to find a method to extract the information of Aβ plaque deposition from MR images and display it. The extraction of Aβ information is essentially a data mining problem, so it is feasible to solve the problem by introducing machine learning methods.

This paper intends to solve this problem using this idea. First, segment the brain regions that possibly contain Aβ plaque deposition on brain MR images. Second, extract the pixels from the brain regions to construct pixel feature vectors (samples). Third, conduct feature selection by maximizing the classification accuracy and obtaining the optimal pixel features. Finally, map the selected pixel features to the corresponding pixels on MR images, thereby showing the Aβ plaque deposition. For detail, please see “Methods”, “Experiment and Discussion” sections.

The remainder of this paper is organized as follows. In “Methods” section, the whole process is introduced to describe how to select the MR image pixels to detect Aβ plaque deposition. In this process, four feature selection algorithms are involved. “Results” and “Discussion” sections describe the experimental results and analysis. “Conclusions” ends the paper and discusses future work. Finally, highlights are presented in last section.

## Methods

### Data description—mouse model and images

The mouse model used was the homozygous APPswe/PS1dE9 double transgenic mouse, bred on a C57BL/6 background. The mice were obtained from Beijing HFK Bio-technology Co., Ltd., Institute of Laboratory Animal Science, Chinese Academy of Medical Science (Beijing, China). These transgenic mice carry human APPswe (Swedish mutations K594N/M595L) and presenilin-1 with exon-9 deletion (PS1-dE9) under control of the mouse prion protein promoter, producing increasing amounts of amyloid deposits in the brain, as well as developing cognitive deficits with age [21]. Female transgenic mice aged 9 months old and their matched, non-transgenic, wild-type (WT) littermates were used in the present study (n = 8, respectively). The animals were housed in temperature- and humidity-controlled rooms with ad libitum access to food and water. All of the research with mice followed the University Policies on the Use and Care of Animals and was approved by the Institutional Animal Experiment Committee of Fourth Military Medical University. All of the experiments were performed blinded with regard to the genetic status of the mice. The mouse model in this paper was the APP transgenic mouse, which causes Aβ plaque deposition. We collected brain MR images from 22 mice (10 with early Alzheimer’s disease [AD] and 12 normal mice [CTL]). The 9 months old App mice are corresponding to the early stage of AD.

The MR images and brain histological images all came from the Beijing animal imaging scan room. The information about the image data is summarized as follows: the MR image sequence was a T2-weighted image sequence (TE first echo), TR: 4000 ms, ETL: 8, and ESP: 10; and the data size was 128 × 128. Each mouse had 12-layer, two-dimensional images (DICOM format). The 4th to 9th slices of brain MR images of each mouse were chosen. Subsequently, a total of 132 two-dimensional images (denoted as samples, 6 × 22 = 132) were obtained, 72 samples of which did not contain Aβ plaque deposition and belonged to normal mice, and 60 samples of which contained Aβ plaque deposition and belonged to mice with dementia. For subsequent pixel feature selection, the 132 samples are randomly divided into three parts. They are training set, validation set and test set respectively. The three parts do not overlap each other. By repeating the division for eight times, the eight groups of the data sets are constructed.

The brains were fixed in 0.01 M PBS (pH 7.4) containing 4 % formalin, dehydrated by a graded series of alcohol, embedded in paraffin and then sectioned into slides 5 μm in thickness. After treatment with 0.1 M formic acid, the brain slices were incubated with beta-amyloid 17–24 (4G8) monoclonal antibody (SIG-39220, Covance, BioLegend, San Diego, California, USA) for 24 h at 4 °C. The slices were then incubated with horseradish-labeled secondary antibody for 1 h, and the color signal was developed with DAB. The nuclei were stained with hematoxylin. The slides were observed, and images were obtained under an Olympus BX53 microscope (Olympus, Japan).

Brain histological images were divided into the immune group and control group, the former containing apparent Aβ plaque deposition. The resolution of the brain histological images was 2560 × 1920, and they were magnified 40 times. Each mouse had six tissue section images including the left and right sections of the hippocampus and the left and right sections of the cerebral cortex.

**Fig. 1:**
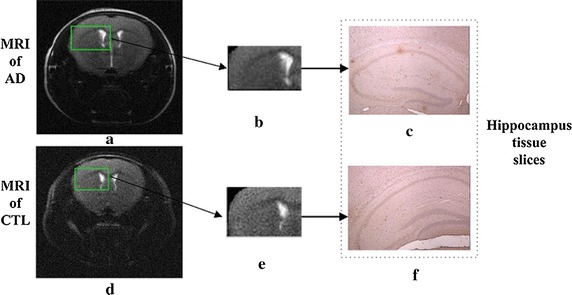
MR and tissue section images of Aβ protein deposition. **a**, **d** MR images of two categories of AD and CTL, from the same slice in each case; **b**, **e** Left hippocampi from two MR images, surrounded by *green boxes*; **c**, **f** The corresponding microscopic images of histology slides

As shown in Fig. 1, the left pictures marked with a and d are MR images of two categories (early AD and CTL); the former contains Aβ plaque deposition, and the latter does not. But there is no obvious difference between the two images. The middle images are the left hippocampi of two MR images, and we cannot distinguish whether they contain Aβ plaque deposition or not. In the figure, the right images are the corresponding microscopy images of histology slides (c and f). By observing the brain histological images, we found that for CTL mice, there was no Aβ plaque deposition, whereas for early lesions in the AD mice, Aβ plaque deposition could be seen in the brain histological slice images (see small brown area).

Past studies have showed the MR image contain the information of the Aβ plaque but cannot show it visually. The result shows that there is strong correlation between the MR pixels and the information of the Aβ plaque. By observation, the histology slices from APP mice (AD) contain the Aβ plaque deposition apparently, but the histology slices from healthy mice (CTL) contain the Aβ plaque barely. Hence there is strong correlation between the information of the Aβ plaque deposition and the classification of CTL and AD. As we known, Aβ plaque deposition is an important prevention and treatment target for AD, so there are strong correlations among the three things. The classification accuracy of the Aβ plaque deposition can be improved by selecting the corresponding MR pixels. The MR pixels can reflect the information of the Aβ plaque deposition. The information of the Aβ plaque deposition can be helpful for improving the classification accuracy of CTL and AD. Therefore, the AD and CTL groups could be considered a gold standard to detect the Aβ plaque deposition. Based on this idea, we could optimize the MR pixels that reflect information about Aβ plaque deposition by maximizing the classification accuracy of AD and CTL samples. Therefore, this idea could be called the detection of Aβ plaque deposition on MR images based on pixel feature selection and class information at the image level.

### Flowchart of the propose algorithm

**Fig. 2:**
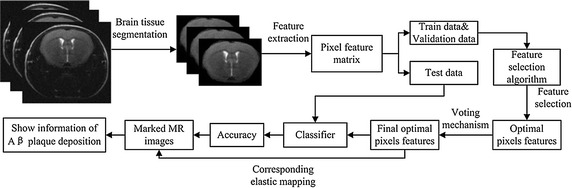
Flowchart of this detection idea. The process consists of four main steps, including segmentation of brain tissue with MRIcro, pixel feature extraction, feature selection with CAGA or PCA, and classifier testing; marking of brain MR images is based on the coordinate information of the pixel features

The flow chart of the proposed method is shown in Fig. 2. First, the brain tissue is segmented manually, and then brain tissue images of the mice are obtained. Second, the pixel values are extracted from the brain tissue images to form feature matrices as data samples. Third, randomly split samples are obtained for training, validation and testing of the three parts, each for training, optimizing, and testing the feature selection and performance of the model respectively. Fourth, the optimal pixel features are obtained by maximizing the classification accuracy of AD. Fifth, the final optimal pixel features are obtained by voting mechanism. Sixth, the test samples are classified as CTL or AD base on the final optimal pixels and the classification accuracy rates are calculated. Finally, elastic mapping is performed of the final optimal pixel features onto the pixels on the MR images of AD, and they are marked to show the location of the Aβ plaque deposition.

### Brain image segmentation

**Fig. 3:**
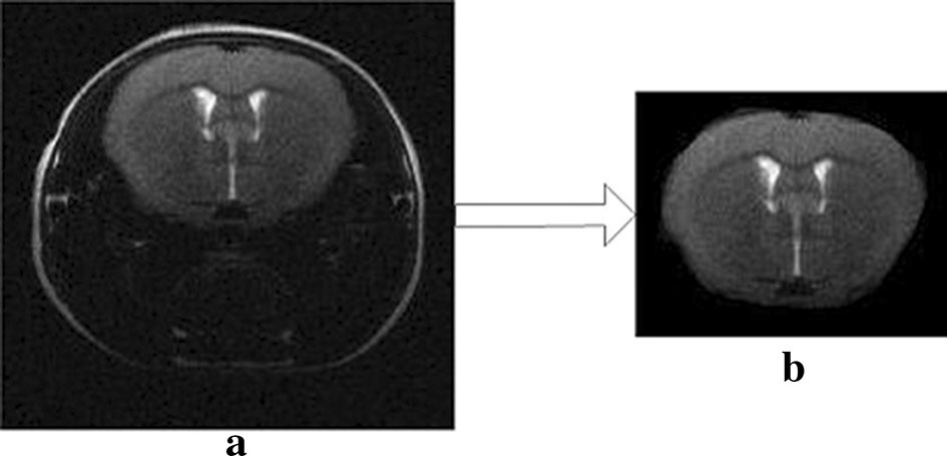
Effect of brain tissue segmentation. **a** The fourth slice of a brain MR image of an AD mouse model. **b** The corresponding segmented brain tissue of the brain MR image

In this paper, MRIcro software was used to examine and transform efficiently the brain MRI of mice and to input and output the brain imaging data. Because Aβ plaque deposition is located in the brain tissue region, the brain tissue region of the mice was the region of interest (ROI). This paper created and preserved the segmented images using MRIcro. To ensure the accuracy of the segmentation, the whole process was conducted under the guidance of a doctor. First, the outline of the brain tissue was manually traced; second, the filling operation was performed; and finally, the ROI was output as an analytic image, as shown in the following Fig. 3b. In this paper, the process of manual extraction of brain tissue images was performed under the supervision of doctors. The segmentation results were accepted by the doctor, and the segmentation accuracy met the requirements.

### Feature extraction

Based on the segmented brain tissue, the gray values of the pixels in the brain tissue were extracted as pixel features. According to different images, the number of pixels in the brain tissue was possibly different, and the lengths of the feature vectors were different. Therefore, based on the shortest feature vector, elastic mapping was conducted of the feature vectors with different lengths onto those with the same length. The length was determined by the shortest feature vector.

### Feature selection

Usually, the feature selection method included three major parts: the feature selection mode, search algorithm, and evaluation criteria. They are described as follows.

#### Feature selection mode

In this paper, two types of feature selection modes were used: wrapper mode and filter mode. The former evaluates subsets of variables, unlike filter approaches, which allow the detection of the possible interactions between variables. Its evaluation criterion is the accuracy rate from the classifier. The latter is an unsupervised learning algorithm that analyzes the internal information of the feature subset to measure its quality.

#### Search algorithm (optimization algorithm)

In the paper, two popular search algorithms were involved here, PCA (principal component analysis) and chain agent genetic algorithm (CAGA algorithm), which the authors proposed previously. The former is a statistical procedure that uses orthogonal transformation to convert a set of observations of possibly correlated variables into a set of values of linearly uncorrelated variables called principal components [22, 23]. The PCA will be described in detail in the ‘PCA’ section.

#### CAGA

Because the former approach has been widely adopted, we describe the principle of the CAGA [24] here. The CAGA algorithm, as an improved agent genetic algorithm, has advantages of high and stable search accuracy and strong robustness. The population individuals are designed to be intelligent agents within this algorithm. Assuming that the agent located in the *j*th node in the *i*th sub-population is expressed as *L*_i,*j*_, the neighborhood domain of *L*_*i*,*j*_ is defined as follows: $Neibor_{i,j} = \left\{ {L_{i,j1} ,L_{i,j2} } \right\}$, where *i* indicates the serial number on the chain agent body, and *j* indicates the *j*th agent on the *i*th chain body.

1 $$L_{i,j1} = \left\{ {\begin{array}{*{20}c} {L_{i,L} } & \quad {j = 1} \\ {L_{i,j - 1} } & \quad {j \ne 1} \\ \end{array} } \right., \quad \quad L_{i,j2} = \left\{ {\begin{array}{*{20}c} {L_{i,1} } & \quad {j = L} \\ {L_{i,j + 1} } & \quad {j \ne L} \\ \end{array} } \right.$$

As seen from the formula above, this chain is closed, and genetic information can spread freely thereon. For details, please see Ref. [24].

**Fig. 4:**
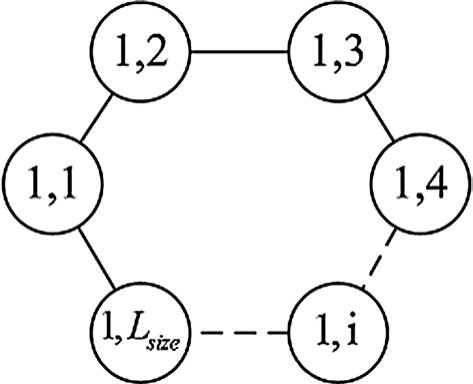
Chain-like agent structure inside the sub-population. The whole *large circle* indicates one population. Each *small circle* represents an agent, and *L*_*size*_ indicates the size of the population. The figure indicates the population structure of the genetic algorithm

The agent ring can be described as that in Fig. 4. Each circle represents an agent, the data in a circle represent its position in the ring, and the agent can interact with the left neighboring position and the right neighboring position.

Energy is defined as follows: an agent, a, represents a candidate solution to the optimization problem in process. The value of its energy is defined as follows:

2 $$Eng(L_{1,i} ) = fitness\left( {L_{1,i} } \right)$$

where *fitness*() indicates the fitness value of some individual in the population. For feature selection, it corresponds to some evaluation criterion.

As seen, each agent represents an individual. To realize the local perceptivity of agents, the environment is constructed as a chain-like structure as mentioned above.

Suppose that the current agent, which is located at $$L_{1,i} = \left( {l_{i,1} ,l_{i,2} , \ldots l_{i,n} } \right)$$$$Max_{1,i} = \left( {m_{i,1} ,m_{i,2} , \ldots ,m_{i,n} } \right)$$, is the agent with maximum energy among the neighbors of *L*_1,*i*_, where *n* indicates the number of genes. *L*_*i*,*n*_ indicates the *n*th gene of the *i*th individual *L*_1,*i*_ (that is, the chromosome), and *m*_1,*n*_ indicates the *n*th gene of *Max*_1,*i*_. That is, $$Max_{1,i} \in Neibors_{1,i}$$ and $$\forall a \in Neibors_{1,i}$$ then $$Eng\left( a \right) \le Eng\left( {Max_{1,i} } \right)$$.

If *L*_1,*i*_ satisfies formula (3), then it persists in the agent chain. Otherwise, it dies, and its chain-point is occupied by *New*_1,*i*_.

3 $$Eng\left( {L_{1,i} } \right) \ge Eng\left( {Max_{1,i} } \right)$$

*Dynamic competition strategy* During the competition process, the $Max_{1,i} = \hbox{max} \left( {L_{{1,i_{1} }} ,L_{{1,i_{2} }} } \right)$. The competition process is performed in ascending order, and after the competition of the 1st agent, the 1st agent is updated. Assuming the *i*th agents before competition and after competition are $L_{1,i}^{pre}$ and $L_{1,i}^{post}$, respectively, so *Max*_1,*i*_ is determined by Eq. (4):

4 $$Max_{1,i} = \left\{ {\begin{array}{*{20}c} {\hbox{max} \left( {L_{{1,L_{size} }}^{pre} ,L_{1,i + 1}^{pre} } \right)} & {i = 1} \\ {\hbox{max} \left( {L_{{1,L_{size} - 1}}^{post} ,L_{1,1}^{post} } \right)} & \quad {i = L_{size} } \\ {\hbox{max} \left( {L_{1,i - 1}^{post} ,L_{1,i + 1}^{pre} } \right)} & {else} \\ \end{array} } \right.$$

##### Dynamic neighborhood competition selection operator

The neighborhood competition selection operator is described as follows: suppose the order of competition selection is from left to right, the current agent is $L_{1,i}^{t}$, and the neighbors are $Nbs_{1,i}$, $Nbs_{1,i} = \left\{ {L_{1,i1}^{t} } \right.\left. {\;\;L_{1,i2}^{t} } \right\},i = 1,2 \ldots \;popsize$. Updating of $L_{1,i}^{t}$ is as follows in Eq. (5):

5 $$\left\{ {\begin{array}{*{20}l} {L_{1,i}^{t} = L_{1,i}^{t} } & \quad {fitness(L_{1,i}^{t} ) > fitness(max(L_{1,i1}^{t} ,L_{1,i2}^{t} ))} \\ {L_{1,i}^{t} = L_{1,i}^{t}\circ L_{1,i1}^{t} } & \quad {(max(L_{1,i1} ,L_{1,i2} ) = L_{1,i1} )\& (fitness(L_{1,i1} ) > fitness(L_{1,i}^{t} ))} \\ {L_{1,i}^{t} = L_{1,i}^{t}\circ L_{1,i2}^{t} } & \quad {(max(L_{1,i1} ,L_{1,i2} ) = L_{1,i2} )\& (fitness(L_{1,i2} ) > fitness(L_{1,i}^{t} ))} \\ \end{array} } \right\}$$

In formula (5), $\circ$ indicates competition selection between agents $L_{1,i}^{t}$ and $L_{1,i1}^{t}$, and the two agents consist of many genes:

6 $$\begin{aligned}L_{1,i}^{t}& = (c_{i,1}^{t} \;c_{i,2}^{t} \ldots c_{i,j}^{t} \ldots c_{i,length}^{t} ), \\ L_{1,i1}^{t} &= (c_{i1,1}^{t} \;c_{i1,2}^{t} \ldots c_{i1,j}^{t} \ldots c_{i1,length}^{t} ) \end{aligned}$$

$c_{i,j}^{t}$ indicates the *j*th gene of $L_{1,i}^{t}$, $c_{i1,j}^{t}$ indicates the *j*th gene of $L_{1,i1}^{t}$, and *length* indicates number of genes of a single agent. The competition selection between agents $L_{1,i}^{t}$ and $L_{1,i1}^{t}$ can be called $L_{1,i}^{t} \circ L_{1,i1}^{t}$, and the process is as follows:

If $c_{i,j}^{t} = c_{i1,j}^{t}$, then $c_{i1,j}^{t}$ does not change; otherwise, if $c_{i,j}^{t} \ne c_{i1,j}^{t}$, then $c_{i,j}^{t} = U(0,1)$, where *U*(0, 1) indicates 0 or 1 randomly selected.

##### Adaptive crossover operator

In the course of the crossover operation, the crossover probability is adaptive. The corresponding formula is given by Eq. (7):

7 $$p_{c} = \left\{ \begin{array}{ll} \left( \frac{{f_{\hbox{max}}^{t}} - {f_{i}^{\prime}}}{{f_{\hbox{max}}^{t}}-{f_{ave}^{t}}}\right)^{\frac{1}{GH(i,i^{\prime})}} &\quad {f^{\prime}} \ge {f_{ave}^{t}} \\ 1& \quad{ f^{\prime}} < {f_{ave}^{t}} \end{array} \right.$$

where $GH(i,i^{{\prime }} )$ is the Hamming distance of $L^{t}_{1,i}$, and $\max_{1,i}$, $\max_{1,i}$ is an individual with a large fitness value in $Nbs_{1,i}$, $f^{{\prime }}$ is a large fitness value within $L^{t}_{1,i}$, and $\max_{1,i}$, $pop^{t - 1}$ is the maximal fitness value among the individuals of this generation, $f_{ave}^{t}$ is the mean fitness value of individuals of this generation. The specific cross-operation is as follows: generate a random number $U(0,1)$ between 0 and 1 and then compare it with *p*_c_ to determine whether $L^{t}_{1,i}$ can cross over with $\max_{1,i}$.The corresponding formula is as follows: $$\left\{ \begin{aligned} cross U(0,1) < p_{c} \hfill \\ uncross U(0,1) \ge p_{c} \hfill \\ \end{aligned} \right.$$. The intersection parents randomly exchange at the same locus, thereby generating new individuals.

##### Adaptive mutation operator

Suppose that the mutation probability is*p*_*m*_, so $$\left\{ \begin{aligned} mutation U(0,1) < p_{m} \hfill \\ unmutation U(0,1) \ge p_{m} \hfill \\ \end{aligned} \right.$$, and generally speaking, *p*_*m*_ is related to the length of the chromosome and is decided by the following equation: $p_{m} = {1/{length}}$.

##### Stopping criterion

To obtain more stable selection results, the paper introduces an adaptive stopping criterion. $f_{ave}$ can reflect the evolution of the current population. *f*_*best*_ indicates the best average fitness value because the beginning. *k*_*stop*_ indicates a counter, which counts the number of *f*_*best*_ that has no change. *N*_*iter*_ indicates the preset maximum iteration number. If *k*_*stop*_ > *k* or if the number of iterations is more than *N*_*iter*_, the search stops.

#### Evaluation criteria

The evaluation criteria are decided based on the feature selection mode. Under the wrapper mode, the evaluation criterion is classification accuracy. The evaluation criterion under the filter mode is characterized by internal information of the feature measurements guidelines. In this paper, we adopt the separability distance criterion.

In this paper, the evaluation criterion under the filter mode is the separability distance criterion, which is one of the classification abilities that characterize the evaluation criteria. Like the mainstream standard to evaluate the separability, the separability criterion could replace classification accuracy for feature selection, and its value is positively proportional to the classification capability. In this paper, the fitness function by the separability distance criterion is designed based on geometric distance. The fitness function is the ratio between the inter-class variance and intra-class variance. The calculation formula is described as follows:

$$\lambda = \frac{{S_{b} }}{{S_{w} }}$$

where $S_{b} = \left( {\bar{c}_{1} - \bar{c}_{2} } \right)^{2}$ indicates inter-class variance and intra-class variance can be expressed as

$$S_{w} = \frac{{P_{1} }}{{N_{1} }}\sum\limits_{i = 1}^{{N_{1} }} {\sum\limits_{k = 1}^{M} {\left( {c_{1ik} - \bar{c}_{1} } \right)}^{2}} + \frac{{P_{2} }}{{M_{2} }}\sum\limits_{j = 1}^{{N_{2} }} {\sum\limits_{k = 1}^{M} {\left( {c_{2jk} - \bar{c}_{2} } \right)} }$$

$P_{1} = \frac{{N_{1} }}{{N_{1} + N_{2} }}$ indicates the ratio of the first type of sample of total sample, while $P_{2} = \frac{{N_{2} }}{{N_{1} + N_{2} }}$ indicates the ratio of the second type of sample of the total sample; *c*_1*ik*_ represents the gray value located in the *k*th column of the *i*th sample within the first class; and *c*_2j*k*_ is the *k*th column’s gray value of the *j*th sample within the second class. $\bar{c}_{1}$ represents the first class center value, and $\bar{c}_{2}$ indicates the second class center value.

#### Classifier—support vector machine (SVM)

Support vector machine (SVM) is currently the mainstream classifier in machine learning [25]. Its main parameters to be trained are described in Eq. (8):

8 $$c = \sum\limits_{q} {a_{q} } k(s_{q} ,x) + b$$

where *c* is the penalty factor, *a*_*i*_ is the learning rate, *q* is the serial number of the sample, *k*() is the kernel, and *b* is the offset. Here, we used linear kernel as the kernel function, which can be written as follows in Eq. (9):

9 $$k(z,z_{c} ) = z^{T} z_{c}$$

where *z* is any point in space, and *z*_*c*_ is the center of the kernel function.

#### Classifier—random forest (RF)

When the random forest algorithm is chosen as the classifier, the corresponding training is conducted as follows [26].

Step 1: Bootstrap methods are used for re-sampling, randomly generating T training sets: $S_{1} ,S_{2} , \ldots ,S_{T}$.
Step 2: Based on each training set, the corresponding decision trees are generated: $C_{1} ,C_{2} , \ldots ,C_{T}$; *m* attributes from M attributes are randomly selected as the splitting attribute set of the current node, and the node is split.
Step 3: Each tree grows integrally, without being pruned.
Step 4: Based on each decision tree, the sample in the test set X is classified, thereby obtaining the corresponding categories, $C_{1} (X),C_{2} (X), \ldots ,C_{T} (X)$.
Step 5: Applying the voting method, the categories of the sample are output by the T decision trees. The category with maximum votes is the final category of the sample.

### Pixel feature selection algorithms

Based on the main parts described above, the four pixel feature selection algorithms are developed for selecting the optimal pixel features, thereby showing the information of the Aβ plaque deposition. The four algorithms are described as follow.

#### GA filter feature selection algorithm (GA_filter)

GA is used for search algorithm. The separability distance criterion (in “Evaluation criteria” section) is used for constructing the fitness function. By genetic iteration, the GA search the optimal pixel features by maximizing the fitness value. The separability distance criterion is a useful criterion for indirectly evaluating the classification accuracy. It has advantage of low time cost and high generalization capability. Here, both the training set and validation set are used for calculating the fitness values based on the feature subset candidates (chromosomes). The feature subset candidate with the highest fitness value is the optimal feature subset or the optimal pixels features (feature vector).

The pseudo code for the GA_filter is shown below:

#### PCA based feature selection algorithm (PCA)

The number of principal components is less than or equal to the number of original variables. This transformation is defined in such a manner that the first principal component has the largest possible variance (that is, accounts for as much of the variability in the data as possible), and each succeeding component, in turn, has the highest variance possible under the constraint that it is orthogonal to the preceding components. The resulting vectors are an uncorrelated orthogonal basis set. The principal components are orthogonal because they are the eigenvectors of the covariance matrix, which is symmetric. By the procedures above, the best components can be obtained. By inverse covariance matrix, the best components can be transformed as the corresponding optimal pixel features. The whole process of PCA is conducted on the training and validation set.

#### GA_SVM wrapper feature selection algorithm (GA_SVM(wrapper))

Different from the GA_filter, the GA_SVM (wrapper) is a kind of wrapper feature selection algorithm. GA is used for search algorithm. The classification accuracy from SVM is used for constructing the fitness function. By genetic iteration, the GA search the optimal pixel features by maximizing the fitness value. The classification accuracy is a useful criterion for directly evaluating the classification accuracy. It has advantage of high classification accuracy. Here, the training set is used for training the SVM with feature subset candidates (chromosomes); the validation set is used for calculating the fitness values based on the trained SVM and the feature subset candidates. The feature subset candidate with the highest fitness value is the optimal feature subset or the optimal features (feature vector).

The pseudo code for the GA_SVM (wrapper) is shown below: 

#### GA_RF wrapper feature selection algorithm (GA_RF(wrapper))

Similar with the GA_SVM (wrapper), the GA_RF (wrapper) is another kind of wrapper feature selection algorithm. GA is used for search algorithm. The classifier is RF rather than SVM. The classification accuracy from RF is used for constructing the fitness function. By genetic iteration, the GA search the optimal pixel features by maximizing the fitness value. Here, the training set is used for training the RF with feature subset candidates (chromosomes); the validation set is used for calculating the fitness values based on the trained RF and the feature subset candidates. The feature subset candidate with the highest fitness value is the optimal feature subset or the optimal features (feature vector).

### Voting mechanism

By the feature selection algorithm, the optimal pixel features can be obtained. By repeating the same feature selection algorithm for m times, the m optimal feature subsets (feature vectors) can be obtained. For each feature, calculate the times *k*_*select*_ that the feature which is selected. If the *k*_*select*_ > *T*_*select*_, then the feature is chosen; else, the feature is not chosen. Usually, the *T*_*select*_ ranges within $\left( {k_{{{{select}/ 2}}} ,k_{{select}} } \right)$.

### Elastic mapping from selected pixel features to the marked pixels on MR images

The main procedure is described as follows:

Step 1: m optimal feature subsets are obtained from the pixel feature selection algorithm by repeating it m times.
Step 2: The voting mechanism is used to obtain the final optimal feature vector.
Step 3: According to the final optimal feature vector, elastic mapping to the original pixels feature vectors that formed in feature extraction section, thereby obtaining the corresponding pixels on the brain MR image.
Step 4: The mapped pixels are marked on the MR image of AD to show the position of the Aβ plaque deposition
Step 5: Steps 1–4 are repeated until all of the MR images of AD are marked.

**Fig. 5:**
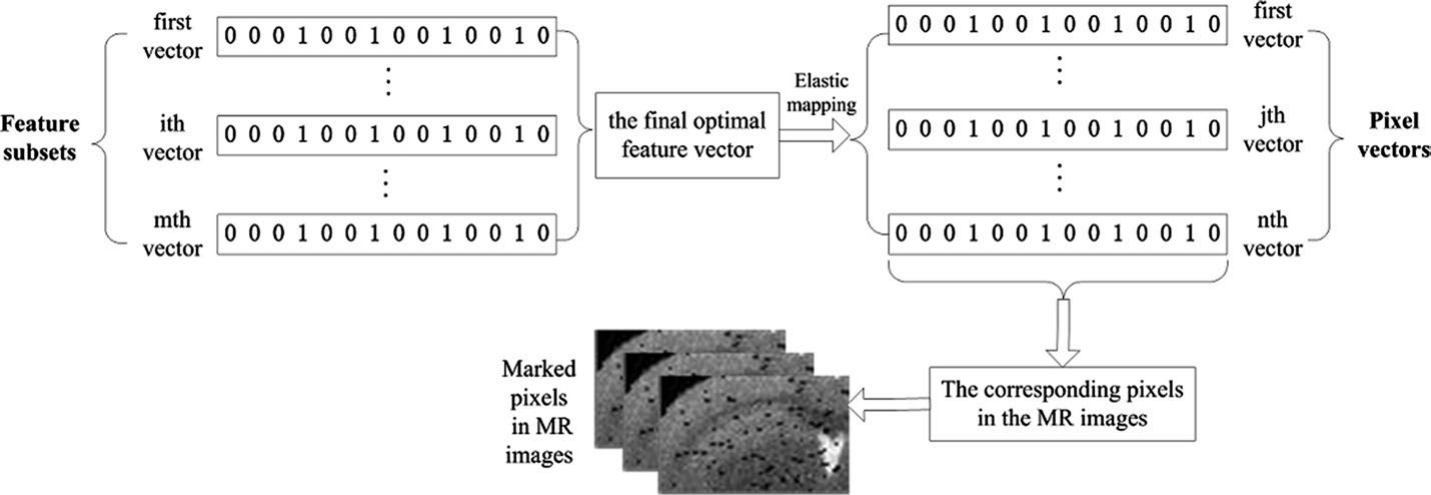
Flowchart of elastic mapping based on the optimal feature subset. “0” indicates that the feature of the location is not selected; “1” indicates that the feature of the location is selected. Feature subsets were obtained by binary coding; m is the total number of samples. By all of the feature subsets, the optimal feature subset is obtained. Pixel vectors with different dimensions were obtained using elastic mapping from the optimal feature vectors. By the pixel vectors, the corresponding pixels in the brain images can be found and marked, thus providing information about Aβ plaque deposition

The process can be seen in Fig. 5.

## Results

### Experimental conditions

The mouse model in the paper consisted of APP transgenic mice, which experience Aβ plaque deposition. We collected brain MR images from 22 mice [10 with early AD, and 12 normal mice (CTL)]. The MR images and all of the brain histological images came from the Beijing animal imaging scan room. The information about the imaging data is summarized as follows: The data were divided into two categories (CTL and AD). The image sequence was a T2-weighted image sequence (TE first echo), TR: 4000 ms, ETL: 8, and ESP: 10; the data size was 128 × 128. Each mouse had 12-layer two-dimensional images (DICOM format). The area of brain tissue on some of the images was small, so these images were removed. The 4th to 9th slices of the brain MR images of each mouse were chosen. Subsequently, a total of 132 two-dimensional images (denoted as samples, 6 × 22 = 132) were obtained, 72 samples of which did not contain Aβ plaque deposition and belonged to normal mice, and 60 samples of which contained Aβ plaque deposition and belonged to mice with dementia.

Pixels in the brain tissue portion of each image sample were extracted, forming a feature vector, with a feature’s value indicating a pixel’s gray value. The shortest feature vector was used as a feature vector template. Every feature vector was aligned (mapped) with the feature vector template. The length of the template vector was 2911, so the images of the 132 samples were converted to gray feature matrices of 2911 × 132, where 132 is the number of data samples, and 2911 is the number of the features.

In this paper, experimental operating system platform was the Windows, version 7, 64-bit operating system, and the memory size was 4 GB. Image extraction of the mice’s brain tissue was performed using an MRIcro medical image viewer, and the data processing was completed in MATLAB, version 2012a.

To verify the accuracy of the algorithms to detect Aβ plaque deposition information on MR images, four feature selection algorithms are involved. Based on the optimal pixels from the feature selection algorithms, the test samples were classified by the corresponding classifiers (SVM, RF). By cross combination, the six kinds of classification experiments were conducted. GA_SVM (filter) means the SVM classifies the test samples based on the optimal MR pixel features from GA_filter. GA_RF (filter) means the RF classifies the test samples based on the optimal MR pixel features from GA_filter. PCA_SVM (filter) means the SVM classifies the test samples based on the optimal MR pixel features from PCA. PCA_RF (filter) means the RF classifies the test samples based on the optimal MR pixel features from PCA. GA_SVM (wrapper) means the SVM classifies the test samples based on the optimal MR pixel features from GA_SVM (wrapper). GA_RF (wrapper) means the RF classifies the test samples based on the optimal MR pixel features from GA_RF (wrapper).

For CAGA, to balance time cost and accuracy better, according to many experimental statistics, we determined the size of the initial population at 50, the initial crossover probability was 0.8, and the initial mutation probability was 0.05. At the end of each iteration, according to the fitness value, we reserved the 50 best individuals, and the maximum number of iterations was set at 30, which helped to find the global optimum quickly and to select the optimal feature subset. The different decision trees in the random forest had impacts on their generalization performance. To reduce the impact of randomness, the paper established 100 forest models randomly and then regarded the average value of the average accuracy rate as the classification accuracy of the current tree. Finally, by considering the random forest containing trees and the modeling speed, we chose the number of trees as the object of optimization and combine it with CAGA to select the optimal feature subset. According to the statistical experiments, 500 was the best number of decision trees for CAGA + random forest. For PCA + random forest, the best number of trees was 650.For voting mechanism, the *k* = 10; *T*_*select*_ = 8.

### Performance evaluation index

The evaluation metrics used to measure the accuracy of whether MRI contained Aβ plaque deposition were the accuracy, sensitivity, and specificity of the test sample. The formula below is the related definition of the classification confusion matrix:

$$Accuracy = \frac{TP + TN}{TP + FP + TN + FN}$$

where *TP* indicates true positives, *TN* indicates true negatives, *FP* indicates false positive, and *FN* indicated false negatives. Sensitivity and specificity are statistical ratios of correctly classified positive and negative instances, respectively:

$$Sensitivity = \frac{TP}{TP + FN}$$

$$Specificity = \frac{TN}{TN + FP}$$

In addition, based on the selected pixel features, the corresponding pixels on the MR images are marked to show the information about Aβ plaque deposition.

### Classification accuracy of AD based on detected Aβ plaque deposition information indirectly

The six kinds of classification experiments discussed above were conducted on the classification of the test samples. Every sample is classified and labeled with AD or CTL class tag. By comparing the labeled class tag of the samples and the real class tag of them, the classification accuracy rates can be calculated. Each experiment was repeated eight times, and the statistical results of the classification are shown in Table 1.

**Table 1:** The classification results under filter and wrapper mode

Evaluation criteria		GA_SVM (filter)	GA_RF (filter)	PCA_SVM (filter)	PCA_RF (filter)	GA_SVM (wrapper)	GA_RF (wrapper)
Accuracy	Average (%)	*73*	65	49	64	71	63
	Best (%)	77	76	58	71	*81*	75
Sensitivity	Average (%)	*75*	70	57	66	64	66
	Best	*81*	78	69	78	70	78
Specificity	Average (%)	68	58	36	60	*75*	56
	Best (%)	70	75	40	60	*88*	70
Selected feature number	Average	1445	1460	125	125	1450	1460
	Best	1470	1435	125	125	1462	1441

**Fig. 6:**
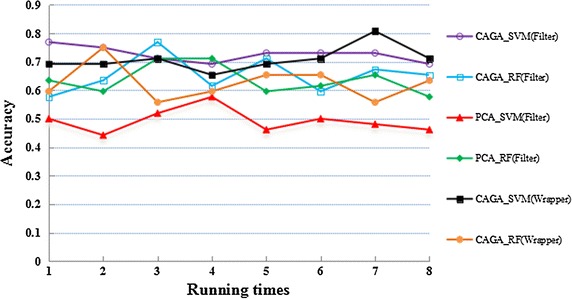
Diagram of classification accuracy of the six types of classification algorithms. The picture describes the average classification accuracy by the six types of classification algorithms for eight repetitions. The *x axis* indicates the running time; the *y axis* indicates the classification accuracy

Most of them were greater than 50 % apparently (see Fig. 6; Table 1), indirectly indicating that the algorithms for detecting Aβ plaque deposition were effective since the Aβ plaque deposition correlate with the classification of AD strongly. In the six experiments, GA_SVM under the filter mode achieved the greatest accuracy of up to 77 %, with an average of 73 % of the accuracy rate, which was possible to form a strong classifier. It would be helpful to improve the accuracy of the current classification methods of AD on MR images. The case is similar with the sensitivity and specificity. Since the App mice slides are corresponding to the early stage of the AD and there is no obvious difference for the MR images of the two categories, the classification accuracy based on one biomarker is applicable.

Figure 6 shows classification accuracy curve of the six experiments repeated eight times. In addition to the accuracy of PCA_SVM (filter), the accuracy rates of the other five experiments were significantly greater than 50 %, which proved that the detection was effective. Under the filter mode, the classification accuracy based on CAGA_SVM was relatively stable; under the wrapper mode, the classification accuracy based on

CAGA_SVM was the greatest, reaching up to 81 % (see Table 1). It was noteworthy that the results based on CAGA_SVM under the filter mode were better than the results under the wrapper mode in the same conditions, which possibly indicates that the separability distance criterion used in the paper performed better.

**Fig. 7:**
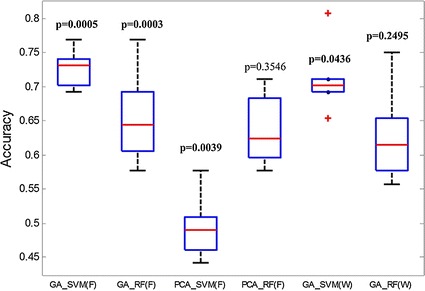
Box plots of six types of algorithms. *F* filter, *W* wrapper. The *six box plots* represent the skewness and tail weight of the data, intuitively and clearly identifying outliers in the data batch. There are two outliers with the algorithm of GA_SVM in wrapper mode. The p value *above* the *box* indicates a significant difference between the algorithms in this paper and a random algorithm

Figure 7 shows the box plots of accuracy for the six experiments. As seen in the figure, we could intuitively find outliers within the data. Observing the length of the box, the top-and-bottom spaced entries, and the length of the whiskers, we could judge the discrete degree and bias of accuracy from the six types of methods. Under the wrapper mode, GA_SVM had two outliers, which indicated that the results had a large range of fluctuations. The GA_SVM under the filter mode with moderate box size and relatively shorter whiskers illustrated that the distribution of the correct rate values was concentrated, so the algorithm was more stable than the other five algorithms.

### Classification performance based on CAGA filter feature selection algorithms

**Fig. 8:**
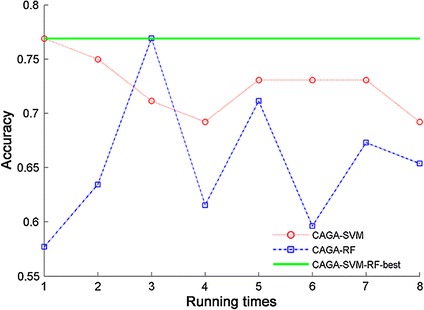
The classification results of the optimal classification algorithms. The* graph* shows the classification accuracy with same feature selection algorithm and with different classifiers. The *green line* indicates the best classification accuracy

Because CAGA + SVM and CAGA + RF in filter mode have best classification accuracy, they are analyzed in this section. Comparisons of the classification results in both cases are shown in Fig. 8.

Compared with RF, the classification accuracy of SVM is far better. The average accuracy is 73 %, and the accuracy rate is 76.92 % (see Fig. 8). In addition, the ACC (accuracy) curve shows that the stability of CAGA_SVM is better, possibly because the SVM is more suitable for the distance separability criterion than RF, and the optimal feature subset obtained by the distance separability criterion is more suitable for training and testing of SVM.

### Analysis of significance level of the feature selection algorithms

**Fig. 9:**
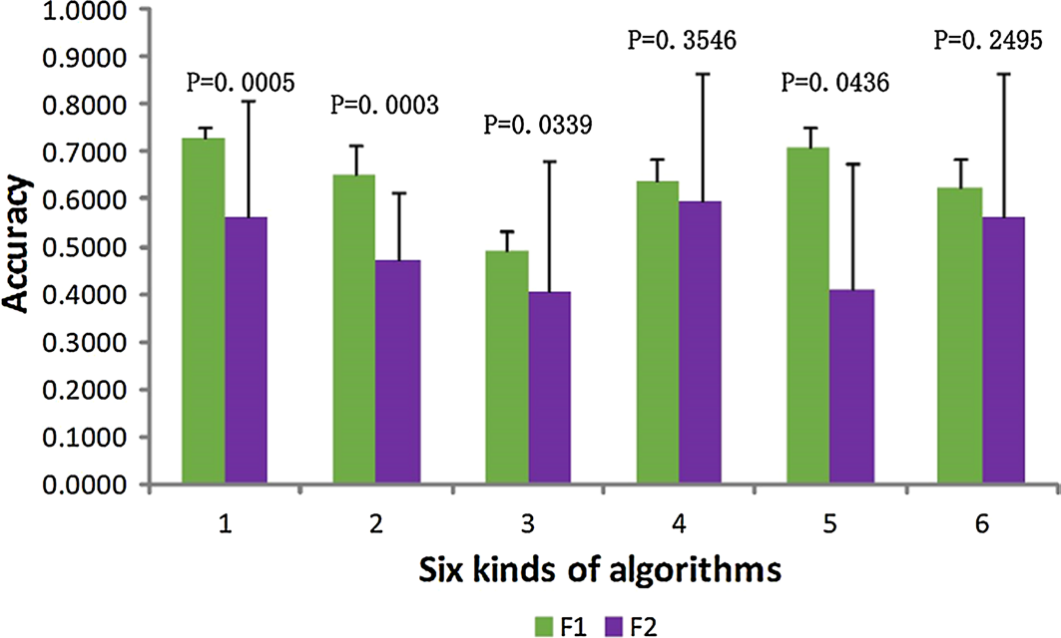
Significant difference between the classification algorithms and the random algorithm. F1, the algorithms on paper; F2, random algorithm; 1, GA_SVM under filter mode; 2, GA_FR under filter mode; 3, PCA_SVM under filter; 4, PCA_RF under filter; 5, GA_SVM under wrapper mode; 6, GA_FR under wrapper mode. p: p value, p < 0.05 indicates that the difference is significant; p > 0.05 indicates that the difference is not significant

To demonstrate the significance level of the classification accuracy of the algorithms proposed in the paper (the proposed algorithms were significantly different from the random pixel selection algorithm), the *t* test of the hypothesis was conducted. The results are shown in Table 2 and Fig. 9.

**Table 2:** Significant analysis of the algorithm

Significant indices	GA_SVM (filter)	GA_RF (filter)	PCA_SVM (filter)	PCA_RF (filter)	GA_SVM (wrapper)	GA_RF (wrapper)
p	*0.0005*	*0.0003*	*0.0339*	0.3546	*0.0436*	0.2495
H	*1*	*1*	*1*	0	*1*	0

H = 0 indicates that the null hypothesis cannot be rejected at the 5 % significance level. H = 1 indicates that the null hypothesis can be rejected at the 5 % level

The four experiments with significant differences in random feature selection were CAGA_SVM (filter), CAGA_RF (filter), PCA_SVM (filter), and CAGA_SVM (wrapper) (see Table 2; Fig. 9). Especially for CAGA_SVM (filter) and CAGA_RF (filter), the p value was far less than 0.001. The results indicated that the high classification accuracy was based on the proposed algorithms themselves, rather than chance. In other words, the results indicated that the effectiveness of detection of the Aβ plaque deposition was based on the proposed algorithms themselves, rather than chance.

### Detection of Aβ protein deposition information in MR images based on pixel feature selection algorithm

According to the Table 1, the classification accuracy of CAGA_filter is best. Therefore, the CAGA_filter is chosen for further study. Conduct feature selection and obtain the optimal pixel feature vector with training set and validation set by CAGA_filter. Repeat the feature selection for ten times, the ten optimal feature vectors are obtained. By voting mechanism, the final optimal feature vector is obtained. Conduct classification of test samples by SVM and the final optimal feature vector (MR pixel features). If the current test sample is classified as AD sample, the sample is labeled and the corresponding pixels in the sample are marked by the elastic mapping and the final optimal feature vector, thereby showing the Aβ plaque depositions. By repeating the experiments for eight times, the statistical classification accuracies are obtained. Please see the Table 3.

**Table 3:** Significance analysis of mapped pixels

	Number of sample sets	1	2	3	4	5	6	7	8	Mean
Number of mapping pixels	Selected pixels by CAGA_filter	311	320	351	333	329	308	321	408	335.13
	Accuracy (%)	77	75	85	69	67	75	71	81	75
	Sensitivity (%)	70	80	75	70	60	75	65	80	72
	Specificity (%)	87	72	91	69	72	75	75	81	78
Significant indices	p	2.68E−51	1.50E−51	3.77E−63	2.21E−54	2.15E−45	2.71E−45	4.27E−53	5.23E−59	6.07E−46
	Corr.	0.0138	0.0013	0.0020	0.0007	0.0005	0.0050	0.0165	0.0192	0.0074

‘Number of mapping pixels’: the number of final optimal pixel features; ‘Number of sample sets’: the No of the groups of test sets; ‘Selected pixels by CAGA_filter’: the pixels selected by the CAGA_filter algorithm; p: p value between the selected pixels by the CAGA_filter and those by the random feature selection algorithm; Corr.: correlation coefficient between the selected pixels by the CAGA_filter and those by the random feature selection algorithm

Seen from the table, the classification performance is improved to some extent compared with that in the Table 1. The mean classification accuracy is 75 % or so. The results mean that the detected Aβ plaque deposition information can be helpful to improve the classification accuracy. Besides, by voting mechanism, the number of the selected pixels decreases greatly. The selected pixels become more stable and can reflect the Aβ plaque deposition information better. In order to show that the classification accuracies are obtained based on the proposed algorithm rather than chance, the significance analysis of the mapped pixels (marked pixels) was conducted. Seen from the p values in the Table 3, all the p values are lower than 0.001 greatly. Apparently, the proposed algorithm is different from random algorithm which randomly selects the MR pixels greatly. The classification accuracy is based on the pixels selected by the CAGA_filter algorithm rather than randomly selected pixels. The classification accuracy indirectly reflects the Aβ plaque information is detected by the final optimal selected pixels. In other words, the proposed detection algorithm is effective.

**Fig. 10:**
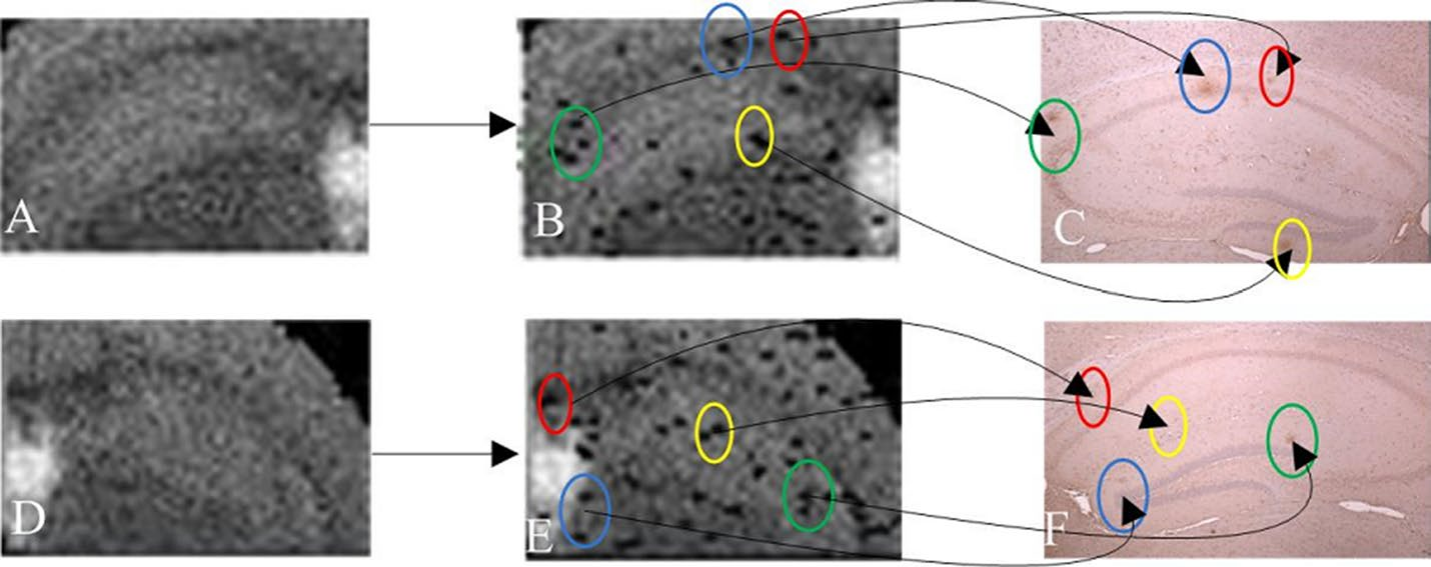
Effect of detection of the Aβ protein deposition. **A**, **D** Hippocampi on brain MR images; **B**, **E** Marked hippocampi of MR images; **C**, **F** Corresponding tissue section images. The regions marked with *different colors* in B and E are related to the distributions of the Aβ plaque deposition in **C** and **F**

According to the coordinates of the final optimal pixel features obtained from CAGA_filter algorithm, the selected pixels could be elastically mapped to the pixels in the MR images of AD from test set, and the mapped pixels are marked to show the Aβ plaque deposition information. Figure 10 shows the marked pixels (detected Aβ plaque deposition information) in the MR images and the corresponding brain histological image slices. A, D: Hippocampi on brain MR images; B, E: Marked hippocampi of MR images; C, F: Hippocampi in corresponding brain histological image slices. The regions marked with different colors in B and E are related to the distributions of the apparent Aβ plaque deposition in C and F.

Seen from the figure, no information about Aβ plaque deposition in hippocampi could be directly seen in image A and D. The brain histology image C and F could show information about Aβ plaque deposition. By the proposed algorithm, the information about Aβ plaque deposition in hippocampi could be shown in MR image B and E.

The different colors of the ellipses show the Aβ plaque depositions which are matched with those marked pixels on the MR image slices. The black line between the same color of the ellipses means the Aβ plaque deposition is matched. Seen from the different colors of the ellipses, the positions of the main Aβ plaque depositions could be shown on image B and E. In other words, the proposed algorithm could detect Aβ plaque deposition on MR images.

By counting the matched and unmatched plaque depositions, the match rate and miss rate can be calculated. The proposed detection algorithm is tested on the eight groups of test samples. The information about the matching of the Aβ plaque deposition can be found in the Table 4 and can show the performance of the proposed algorithm. The match rate = (number of matched Aβ plaque depositions)/(total number of the Aβ plaque depositions); the miss rate = (number of unmatched Aβ plaque depositions)/(total number of the Aβ plaque depositions).

**Table 4:** Information about the matching of the Aβ plaque by the proposed detection algorithm

No. of groups of test sets	Number of CTL samples	Number of AD samples	Sum of Aβ plaques in hippocampus	Number of matched Aβ plaques	Match rate (%)	Number of unmatched Aβ plaques	Miss rate (%)
1	32	20	153	144	94	9	6
2	32	20	167	149	89	18	11
3	32	20	189	176	93	13	7
4	32	20	142	133	94	9	6
5	32	20	139	125	90	14	10
6	32	20	168	152	90	16	10
7	32	20	167	147	88	20	12
8	32	20	159	148	93	11	7

No. of groups of test sets: the sequence number the eight groups of the test sets; Number of CTL samples: the number of the CTL samples in each group of data set; Number of AD samples: the number of the AD samples in each group of data set; sum of Aβ plaque depositions in hippocampus: the total number of the Aβ plaque depositions in hippocampus in the brain histological image slices of AD; Number of matched Aβ plaque depositions: the total number of the Aβ plaque depositions which are matched by the marked pixels in brain MR images of corresponding AD samples; match rate: the ratio of the number of the matched Aβ plaque depositions to total number of the Aβ plaque depositions; Number of unmatched Aβ plaques: the total number of the Aβ plaque depositions which are not matched by the marked pixels in brain MR images of corresponding AD samples; miss rate: the ratio of the number of the unmatched Aβ plaque depositions to total number of the Aβ plaque depositions

Seen from Table 4, for every group of test set, there are 20 AD samples. Since the test sets are constructed by random division of whole data set, there are different slices from different mice in every test set. With the AD MR samples, the corresponding brain histological image slices can be found. By observing the brain histological image slices, the apparent Aβ plaque depositions can be found and counted with the help from clinician of neurology. By summing up the Aβ plaque depositions in the 20 slices, the total number of the Aβ plaque depositions in hippocampus in one group of test set can be calculated. Seen from the number of the eight groups of test sets, the numbers are similar and range between [139, 189]. The one possible reason is that all the AD mice have the same age (9 month old).

By comparing the Aβ plaque depositions in hippocampus in the brain histological image slices and the marked pixels in the corresponding brain MR image slices, the matched and unmatched Aβ plaque depositions can be found. By accumulating them, the number of matched and unmatched Aβ plaque depositions in hippocampus can be obtained. The match rate and miss rate can be obtained. Seen from the rates, most of them are over 90 %. The results show that the proposed detection algorithm can detect most of the Aβ plaque depositions while no other method can do the thing before. According to the relevant theory about the Aβ plaque deposition of AD, the Aβ plaque deposition is positively proportional of the progress of AD, but the volume and the distribution of the Aβ plaque deposition do not correspond to the different states of AD one-to-one strictly. Hence, the 80–90 % of match rate to show the apparent Aβ plaque deposition can be acceptable.

In order to further to verify the performance of the selected MR pixels (detected Aβ plaque deposition), ‘Mean total intensity values in every AD samples’ and ‘Mean total intensity values in every CTL samples’ are calculated for comparison. First, based on the selected MR pixels, the intensity values of the selected pixels in every sample are found and accumulated. Second, in each group of data sets, the total intensity values of the 20 AD samples (called ‘TI_AD values’) and the 32 CTL samples (called ‘TI_CTL values’) are obtained respectively. Third, mean intensity values of 20 AD samples (called ‘MTI_AD values’) are obtained (MTI_AD values = TI_AD values/20); mean intensity values of 32 CTL samples (called ‘MTI_CTL values’) are obtained (MTI_CTL values = TI_CTL values/32). The relevant information can be found in Table 5.

**Table 5:** Classification capability of selected MR pixels

No. of groups of data sets	1	2	3	4	5	6	7	8	Mean value	p value
Mean total intensity values in every AD samples (MTI_AD, ×10^6^)	6.15	5.78	6.06	6.22	5.72	5.76	5.86	5.69	5.91	<0.001
Mean total intensity values in every CTL samples (MTI_CTL, ×10^6^)	9.10	8.52	9.71	8.80	8.68	9.03	8.49	9.50	8.98	

‘No. of groups of data sets’: the No. of the groups of test sets; ‘Mean total intensity values in every AD samples’: mean value of the sum of intensity values of the selected pixels within one image slice of AD; ‘Mean total intensity values in every CTL samples’: mean value of the sum of intensity values of the selected pixels within one image slice of CTL; Mean value: the mean value of the MTI_AD values of the eight groups of data sets and the mean value of the MTI_CTL values of the eight groups of data sets; p_value: significant difference between the MTI_AD values and MTI_CTL values

Seen from Table 5, for every group of data sets, the MTI_AD value always is lower than MTI_CTL value. The mean value of the MTI_AD values is lower than the MTI_CTL values too. The results show that the intensity value based on the selected MR pixels can distinguish the image slices of AD and CTL. The p value is lower than 0.001 greatly. The results show that the difference between the MTI_AD values and MTI_CTL values are apparent. The classification based on the selected MR pixels is stable and reliable. By considering the AD with Aβ plaque deposition and the CTL without Aβ plaque deposition, the results indirectly support the conclusion that the selected MR pixels reflect the information of the Aβ plaque deposition.

## Discussion

In this work, we proposed a detection algorithm for showing Aβ plaque deposition on MR images. First, the brain tissue is segmented manually, and then brain tissue images of the mice are obtained. Second, the pixel values are extracted from the brain tissue images to form feature matrices as data samples. Third, randomly split samples are obtained for training, validation and testing of the three parts, each for training, optimizing, and testing the feature selection and classification of the model. Fourth, the optimal pixel features are obtained by maximizing the classification accuracy. Fifth, the final optimal pixel features are obtained by voting mechanism. Sixth, the test samples are classified as CTL or AD base on the final optimal pixels and the classification accuracy rates are calculated. Finally, elastic mapping is performed of the optimal pixel features onto the pixels on the MR images of AD, and they are marked to show the location of the Aβ plaque deposition.

There are two types of mouse models—CTL and AD: AD model contains Aβ plaque deposition, and CTL model does not. The result shows that there is strong correlation between the information of the Aβ plaque deposition and the classification of CTL and AD.

We verified the effectiveness of the proposed detection algorithm by the following experiments:

1. Based on the optimal pixels from the four feature selection algorithm, the test samples were classified by the corresponding classifiers (SVM, RF). By cross combination, the six kinds of classification experiments were conducted. All the average classification accuracies of the six kinds of classification experiments are above 50 % greatly. The best classification accuracy can achieve the 80 %.
2. According to the classification accuracies, the GA_SVM (filter) is best. Therefore, the GA_fiter is further studied. By repeating the GA_filter on the training and validation samples, the k optimal MR pixel feature vectors are obtained. By voting mechanism, the final optimal MR pixel feature vector is obtained. Based on the final optimal MR pixel feature vector, SVM is used to classify the test samples, label them, and output the classification accuracies. The classification accuracy is improved further.
3. With the final optimal MR pixels (MR pixel feature vector), the test samples of AD are marked with the selected MR pixels by elastic mapping, thereby showing the Aβ plaque deposition in the MR samples visually. By comparing the marked MR pixels and the Aβ plaque depositions in the corresponding brain histological image slices by clinician, most of the apparent Aβ plaque depositions are matched by the marked MR pixels. The results can directly support the effectiveness of the proposed feature selection algorithm.
4. Based on the matched Aβ plaque deposition, the match rate and miss rate were calculated. They are satisfying and meet the elementary requirement from the department of neurology in hospital. The experimental results of the significance level of the propose detection algorithm show that the positive results are stable and reliable rather than by chance.
5. The classification capability of the selected MR pixels by the propose algorithm is studied and shown. The experimental results show that the selected MR pixels can distinguish the CTL and AD samples significantly.

To the best knowledge of the authors, the detection of Aβ plaque deposition from brain MR images alone has not been discussed before in public. Although PET can detect Aβ plaque deposition with a specific tracer to some extent, it essentially has low resolution and data volume and cannot provide information about anatomical structures and small lesions, which are very important for the diagnosis of the AD, as the “Background” section discussed. Furthermore, PET is radioactive and expensive, preventing it from clinical application and from being accepted by patients requiring detection. In comparison, MRI is inexpensive, noninvasive and non-radioactive, and it can provide information about anatomical structures and small lesions. If it can detect Aβ plaque deposition, it will be a better imaging technology for clinical application. This paper proved that it is feasible to detect information on Aβ plaque deposition with MR images alone, providing a solution for related research.

Because the detection of Aβ plaque deposition using only brain MR images has not been discussed before, no existing methods were compared with the method proposed in this paper. To show that the idea of detecting Aβ plaque deposition on brain MR images alone is feasible, six pixel selection and classification experiments were realized. According to the experimental results, most of the data show that the idea is feasible. In addition, the classification rate was as high as 80 %, with high significance level, indicating that the idea of pixel selection based on the classification of images was feasible and reliable.

The best feature selection algorithm is chosen from the four feature selection algorithms. Based on the selected pixels and voting mechanism by it, the final optimal selected pixels are obtained. By elastic mapping, the corresponding pixels on brain MR images of AD were found and marked to show Aβ plaque deposition. By comparing the marked pixels on brain MR images of AD and the Aβ plaque deposition on corresponding histology images of the brain, it was found that the major Aβ plaque deposition was not missed. Compared with the random algorithm, this proposed idea and the proposed algorithm were effective with a high significance level. The hit rate and miss rate were calculated and support the effectiveness of the proposed detection algorithm of the Aβ plaque deposition on MR images.

## Conclusions

Aβ plaque deposition is an important target for early AD diagnosis and the evaluation of treatment, so the noninvasive and nonradioactive detection of Aβ plaque deposition is necessary, especially for real applications. MRI is a safe and cost-effective imaging method, and can contain information about Aβ plaque deposition. However, it cannot detect the Aβ plaque deposition directly and there is currently no existing method to extract Aβ plaque deposition information and to show it on MR images.

In order to solve this problem, this paper proposed MR pixel feature selection algorithm to search for the Aβ plaque deposition information on MR images by maximizing the classification accuracy of AD and CTL MR samples. The experimental results showed that the algorithms in the paper could obtain the best classification accuracy of CTL and early AD more than 80 % with high significance. In addition, the selected pixels could show the position of Aβ plaque deposition with high match rate. Most of the main Aβ plaque deposition was not missed almost. The selected pixels can help to distinguish the CTL and early AD samples significantly.

The proposed detection method is based on the class information of the image slices, and they can detect Aβ plaque deposition based on class information at the image level. Although it is effective to detect the Aβ plaque from brain MR images, there are many works to do to further evaluate and refine the proposed detection algorithm in the future. For example, more mice models possible are needed; human body experiments are needed for clinical research; it is useful to construct a diagnosis algorithm of AD by combing the detected Aβ plaque information with the other MR biomarkers.

## Highlights

This paper proposed pixel feature selection algorithm as detection algorithm to extract information of Aβ plaque deposition from MR images by maximizing classification accuracy. The main contributions and innovations of this paper can be described as follows:

1. This paper proposed a detection algorithm of Aβ plaque deposition on MR images, and the effectiveness of the algorithm was verified.
2. The detection of Aβ information was transformed into a classification problem, thereby making the detection easy.
3. This paper proposed MR pixel feature selection algorithm as detection algorithm to realize the detection of the Aβ plaque deposition on MR images by maximizing the classification accuracy of AD.
4. Since the selected MR pixels can match the Aβ plaque deposition well, the detected Aβ plaque deposition can help the clinicians and the researchers observe the Aβ plaque deposition on MR images. By considering the advantages of the MRI, the function can be helpful for putting the Aβ into clinical applications.
5. The detection of the Aβ plaque deposition on MR images can be helpful for constructing the detection of the Aβ plaque deposition with multiplex-model imaging by combining the PET.
6. Since all the AD mice models have same age (9 months old), the non-invasively detected Aβ plaque deposition information has potential to be helpful for measuring the Aβ plaque deposition of the mice in vivo in this time point or any time point. The function is helpful for better understanding the mechanism of the occurrence and development of the Aβ plaque deposition of mouse model in vivo.

## Abbreviations

Aβ: amyloid β-protein
AD: Alzheimer’s disease
CTL: control
MRI: magnetic resonance imaging
SP: senile plaques
PET: positron emission computed tomography
WT: wild-type
CAGA: chain agent genetic algorithm
SVM: support vector machine
RF: random forest
